# A Pioneering Study Assessing Knowledge and Awareness of Sickle Cell Disease and Its Association with Oral Health Among Pediatric and General Dentists in Madhya Pradesh, India

**DOI:** 10.7759/cureus.92486

**Published:** 2025-09-16

**Authors:** Vaishnavi Parihar, Debapriya Pradhan, Nimisha Paul, Saurabh Tiwari, Raksha Thakur, Swarnika Parihar, Vaishnavi Gupta, Kartik Sinha, Anaghaa G Menon, Aakrati Vishwakarma

**Affiliations:** 1 Department of Pedodontics and Preventive Dentistry, Hitkarini Dental College and Hospital, Jabalpur, IND; 2 Department of Biochemistry, Hitkarini Dental College and Hospital, Jabalpur, IND; 3 Department of Pedodontics and Preventive Dentistry, Career Post Graduate Institute of Dental Sciences and Hospital, Lucknow, IND; 4 Department of Oral Medicine and Radiology, Hitkarini Dental College and Hospital, Jabalpur, IND; 5 Department of Pedodontics and Preventive Dentistry, People's College of Dental Sciences and Research Centre, Bhopal, IND; 6 Department of Pedodontics and Preventive Dentistry, Modern Dental College and Research Centre, Indore, IND

**Keywords:** hemoglobin s, prophylactic antibiotics, pulp necrosis, sickle cell disease (scd), vascular occlusion

## Abstract

Introduction

The region of Central India, particularly Madhya Pradesh, is characterized by a high incidence of sickle cell disease (SCD) affecting diverse demographic groups. Given the intricate relationship between SCD and oral health, this research aims to assess the understanding and awareness of dentists in Madhya Pradesh, focusing on both pediatric and general dental practitioners.

Methods

A cross-sectional online survey was conducted using a structured, self-administered questionnaire (Google Forms, Google Inc., Mountain View, CA) comprising 22 closed-ended questions. The questionnaire was disseminated to 200 dental professionals, including 100 pediatric dentists and 100 general dentists practicing in Madhya Pradesh, via WhatsApp groups (Meta Platforms Inc., Menlo Park, CA) of dental associations and personal networks. The gathered data underwent analysis via IBM SPSS Statistics software, version 23.0 (IBM Corp., Armonk, NY), revealing statistical patterns and correlations.

Results

A significant knowledge gap was observed between pediatric dentists and general dentists in Madhya Pradesh regarding the oral health consequences associated with SCD. While 84% of pediatric dentists recognized the association between SCD and oral health, only 30% of general dentists demonstrated correct knowledge. General dentists showed limited understanding in areas such as SCD testing timing (36%), bone marrow transplant as a cure (29%), common oral manifestations (41%), pulp necrosis in healthy teeth (24%), and local anesthesia safety (33%). However, both groups exhibited good knowledge of aspirin contraindications and prophylactic antibiotic use.

Conclusion

While pediatric dentists showed satisfactory knowledge, general practitioners require supplementary education to improve their awareness and management of SCD. Future studies should expand to multiple states, use objective measures, and include larger, random samples to enhance generalizability.

Clinical significance

This pioneering study in Madhya Pradesh and India highlights the need for enhanced education on SCD among dental practitioners. It emphasizes training on SCD diagnosis, oral features, and patient management to improve dental care for SCD patients in the region.

## Introduction

Sickle cell disease (SCD) is a genetic condition marked by red blood cell abnormalities, affecting their form and function. Key clinical manifestations include hemolytic anemia and vascular occlusion, significantly impacting patient health. There is a mutation that substitutes valine for glutamic acid in the beta-globin chain, leading to abnormal hemoglobin S production. When oxygen levels drop, red blood cells containing hemoglobin S become misshapen, taking on a distinctive sickle shape [1]. Sickle-shaped red blood cells survive for only 12 to 17 days, a marked deviation from the typical 120-day lifespan of healthy red blood cells, resulting in compromised blood flow and oxygenation of body tissues [2]. They can block small capillaries, leading to vaso-occlusion, which impedes blood flow and causes tissue damage. This blockage can result in various complications and painful episodes [3].

SCD is associated with a range of complications, affecting both overall health and oral well-being. Systemic complications can be acute, such as stroke, acute chest syndrome, severe infections, and debilitating bone pain, or chronic, including organ damage and dysfunction. SCD often presents with various oral lesions, including pulp necrosis, mucosal damage from anemia, fungal infections linked to antibiotics, delayed dental eruption, bone pain, maxillary osteomyelitis, and neuropathies affecting nerves like the mental nerve in the chin [4]. Recognizing the numerous oral manifestations of SCD is crucial, even if some are non-specific, as understanding the disease process enables effective oral healthcare management and improves patient care [5].

The worldwide burden of SCD saw a 41.4% surge over the period of 2000-2021, affecting 7.74 million people compared to 5.46 million. In India, the disease disproportionately affects tribal communities, with Madhya Pradesh, India, being a significant hotspot. The prevalence of sickle hemoglobin in the state ranges from 10% to 33%. A significant proportion (around 75%) of the state's sickle cell anemia burden is localized to four tribal districts: Alirajpur, Anuppur, Chhindwara, and Dindori, according to the 2020-2021 annual report [6, 7]. The Government of India's Ministry of Health and Family Welfare has flagged SCD as a major health concern affecting tribal communities. In a bid to combat SCD, Prime Minister Narendra Modi launched the National Sickle Cell Anaemia Elimination Mission (NSCAEM) from Shahdol, Madhya Pradesh, in July 2023, marking a significant step towards eliminating this genetic disorder as a public health problem by 2047 through prevention and management strategies [8].

This study seeks to assess the awareness of pediatric and general dentists in Madhya Pradesh about the relationship between oral health and SCD, aiming to identify knowledge gaps in recognizing oral signs essential for early detection and furthering national efforts to eradicate the condition.

## Materials and methods

Research design and sampling

This study was conducted at Hitkarini Dental College and Hospital, Jabalpur, India. The study employed a quantitative research approach, utilizing a cross-sectional design to investigate the knowledge and awareness of dental professionals in Madhya Pradesh regarding SCD and its impact on oral health. A combination of purposive and snowball sampling techniques was employed to recruit the participants. A sample of 200 dental professionals, comprising 100 pediatric dentists and 100 general dentists in Madhya Pradesh, was purposefully selected based on inclusion criteria, and further participation was facilitated through professional WhatsApp groups (Meta Platforms Inc., Menlo Park, CA) and peer sharing within personal networks. The sample size, determined using a pilot study's response rate, 95% confidence interval (CI), and 5% absolute precision, was 90 dentists per group. Accounting for potential non-response and incomplete responses, the sample size was increased to 100 dentists per group, representing a 10% increment.

Inclusion criteria

The study included dental professionals who met specific requirements, including registration as pediatric or general dentists, current practice in Madhya Pradesh, provision of informed consent, and submission of fully completed questionnaires. This ensured that the participants were qualified, relevant to the study's geographic scope, and provided meaningful data.

Exclusion criteria

Dental professionals were excluded from the study if they lacked registration as pediatric or general dentists, practiced outside Madhya Pradesh, did not provide informed consent, or submitted incomplete questionnaires.

Data collection instrument

A structured, self-administered questionnaire was developed to collect data from the study participants using Google Forms (Google Forms, Google Inc., Mountain View, CA). The questionnaire consisted of 22 closed-ended questions, divided into four sections: demographic information, awareness about the association of SCD with oral health and the NSCAEM, basic knowledge of SCD, and knowledge of SCD-related oral features and dental management strategies (Appendix A). The questionnaire was pretested among a small group of dental professionals to ensure its validity and reliability.

Validation of the questionnaire

The questionnaire's validity was assessed through two key methods, which were as follows: (1) Face validity assessment: Following pilot testing, participants provided feedback on the questionnaire's acceptability, length, language, clarity, and practicality. More than 90% of respondents found the questionnaire to be straightforward and user-friendly. (2) Content validity and reliability evaluation: A panel of academic experts reviewed the questionnaire's content, yielding a mean content validity ratio of 0.83. Cronbach's alpha values, measuring internal consistency, ranged from 0.71 to 0.86 across sections C and D, indicating acceptable to good reliability.

Data collection procedure

The questionnaire was disseminated to the study participants through WhatsApp groups of dental associations and personal networks. This approach ensured a targeted reach and facilitated data collection from a geographically dispersed sample. Participants were informed about the purpose and scope of the study, and their consent was obtained before they completed the questionnaire. The data obtained was entered in Microsoft Excel 365 (Microsoft Corp., Redmond, WA).

Data analysis

The collected data were analyzed using a robust statistical software package, IBM SPSS Statistics software, version 23.0 (IBM Corp., Armonk, NY). Descriptive statistics, including frequencies, percentages, and means, were calculated to summarize the data. Inferential statistics, such as chi-square tests and Pearson's chi-square test, were used for comparisons between groups. Statistical significance was set at p < 0.05.

The analysis revealed valuable insights into the knowledge and awareness of dental professionals in Madhya Pradesh regarding SCD and its oral health implications.

## Results

Among the total 200 study subjects, 146 (73%) belonged to the age group 26-35 years, 42 (21%) belonged to the age group 36-45 years, and 12 (6%) belonged to the age group 46-55 years. There were 62 (31%) males and 138 (69%) females. There were equal numbers of pediatric dentists and general dentists, i.e., 100 (50%) in each group. According to the years of clinical experience, 93 (46.50%) had less than five years, 67 (33.50%) had five to 10 years, and 40 (20%) had more than 10 years of experience. Respondents' biographic and demographic details are summarized in Table 1.

**Table 1:** Demographic details of the study subjects

Demographic Characteristics	N	%
Age groups		
26-35 years	146	73
36-45 years	42	21
46-55 years	12	6
Gender		
Male	62	31
Female	138	69
Type of practice		
Pediatric dentist	100	50
General dentist	100	50
Years of clinical experience		
< 5 years	93	46.50
5-10 years	67	33.50
> 10 years	40	20

Data on participants' understanding of the connection between SCD and oral health were organized into a table, with percentage calculations presented alongside, as illustrated in Table 2.

**Table 2:** Comparison of knowledge and awareness about sickle cell disease between pediatric dentists and general dentists

S.No.	Question	Response	Pediatric Dentist n (%)	General Dentist n (%)	Chi-square Test	P-value	Significance
1	Is sickle cell disease (SCD) associated with oral health?	Yes	84 (84)	30 (30)	χ² = 61.668, df = 2	P = 0.000	Very highly significant
		No	02 (2)	26 (26)	—	—	—
		Maybe	14 (14)	44 (44)	—	—	—
2	Awareness of the National Sickle Cell Anemia Elimination Mission	Yes	58 (58)	56 (56)	χ² = 0.082, df = 1	P = 0.775	Not significant
		No	42 (42)	44 (44)	—	—	—
3	Is SCD an inherited blood disorder?	Correct (Yes)	100 (100)	100 (100)	Not applicable	—	—
		Incorrect	00 (0)	00 (0)	—	—	—
4	Lifespan of red blood cells (RBCs) in SCD	Correct (10–20 days)	52 (52)	46 (46)	χ² = 0.720, df = 1	P = 0.396	Not significant
		Incorrect	48 (48)	54 (54)	—	—	—
5	The most appropriate time to test SCD in a child	Correct (Newborn screening)	71 (71)	36 (36)	χ² = 24.621, df = 1	P = 0.000	Very highly significant
		Incorrect	29 (29)	64 (64)	—	—	—
6	Can SCD be diagnosed using a simple blood test?	Correct (Yes)	68 (68)	66 (66)	χ² = 0.090, df = 1	P = 0.764	Not significant
		Incorrect	32 (32)	34 (34)	—	—	—
7	SCD can be cured by?	Correct (Bone Marrow Transplant)	65 (65)	29 (29)	χ² = 26.014, df = 1	P = 0.000	Very highly significant
		Incorrect	35 (35)	71 (71)	—	—	—
8	Common oral manifestations in SCD children	Correct (Multiple listed)	77 (77)	41 (41)	χ² = 26.788, df = 1	P = 0.000	Very highly significant
		Incorrect	23 (23)	59 (59)	—	—	—
9	Pulp changes in SCD	Correct (Pulp necrosis in healthy teeth)	62 (62)	24 (24)	χ² = 29.457, df = 1	P = 0.000	Very highly significant
		Incorrect	38 (38)	76 (76)	—	—	—
10	Commonness of oral fungal infections in SCD	Correct (Yes)	74 (74)	53 (53)	χ² = 9.514, df = 1	P = 0.002	Highly significant
		Incorrect	26 (26)	47 (47)	—	—	—
11	Radiographic bone changes in SCD	Correct (Yes)	78 (78)	69 (69)	χ² = 2.079, df = 1	P = 0.149	Not significant
		Incorrect	22 (22)	31 (31)	—	—	—
12	Prophylactic antibiotics before surgery in SCD	Correct (Recommended)	81 (81)	67 (67)	χ² = 5.094, df = 1	P = 0.024	Significant
		Incorrect	19 (19)	33 (33)	—	—	—
13	Contraindicated analgesic in SCD	Correct (Aspirin)	71 (71)	65 (65)	χ² = 0.827, df = 1	P = 0.363	Not significant
		Incorrect	29 (29)	35 (35)	—	—	—
14	Contraindication of local anesthesia in SCD	Correct (Not contraindicated)	57 (57)	33 (33)	χ² = 11.636, df = 1	P = 0.001	Highly significant
		Incorrect	43 (43)	67 (67)	—	—	—
15	Use of anxiolytics/sedation in SCD	Correct (Yes)	66 (66)	45 (45)	χ² = 8.928, df = 1	P = 0.003	Highly significant
		Incorrect	34 (34)	55 (55)	—	—	—
16	Importance of preventive dental care in SCD	Correct (Yes)	96 (96)	94 (94)	χ² = 0.421, df = 1	P = 0.516	Not significant
		Incorrect	04 (4)	06 (6)	—	—	—

A substantial proportion of pediatric dentists (84%) compared to general dentists (30%) recognized the link between SCD and oral health outcomes, indicating a marked difference in awareness of the intricate relationship between the condition and dental well-being among these two groups of dental professionals (Cohen's h = 1.16, signifying a large effect size).

Similar proportions of pediatric dentists (58%) and general dentists (56%) were aware of the NSCAEM, indicating comparable knowledge levels between the two groups (Cohen's h: 0.04). This awareness is crucial in enabling them to provide patient-centered care and services for those affected by SCD.

Pediatric dentists (71%) were more likely than general dentists (36%) to identify newborn screening as optimal for SCD testing (Cohen's h = 0.72), likely reflecting the differences in training and clinical exposure that could influence pediatric care outcomes.

A notable knowledge gap exists between pediatric dentists (65%) and general dentists (29%) regarding bone marrow transplant as a potential cure for SCD, with a significant effect size difference (Cohen's h = 0.74).

Pediatric dentists (77%) were almost twice as likely as general dentists (41%) to accurately recognize common oral manifestations of SCD in children, underscoring differences in awareness between these two groups (Cohen's h = 0.75).

A substantial disparity exists between pediatric dentists (62%) and general dentists (24%) in recognizing the risk of pulp necrosis in clinically sound teeth among SCD patients (Cohen's h = 0.79), potentially influencing the timely diagnosis and management.

Pediatric dentists and general dentists exhibit differing knowledge levels in managing SCD patients. While both groups demonstrate awareness of prophylactic antibiotics (81% vs 67%, Cohen's h = 0.32) and aspirin avoidance (71% vs 65%, Cohen's h = 0.13), pediatric dentists are notably more knowledgeable about local anesthesia safety (57% vs 33%, Cohen's h = 0.49), highlighting an area for further education among general dentists.

## Discussion

SCD can have profound effects on oral health, making it crucial for dental professionals to have a solid grasp of the condition [3]. SCD can significantly impact oral health, manifesting in various dental and oral complications. Although these manifestations are often non-specific, dentists can play a crucial role in identifying potential cases [4, 9].

Tribal communities in India bear a significant brunt of SCD, and Madhya Pradesh stands out as a region with a high disease burden. The NSCAEM seeks to enhance patient care and reduce disease prevalence through a multifaceted strategy encompassing screening, awareness, and comprehensive management. This can be achieved by counseling the patients for POCT and referring them for hematological testing, ultimately supporting the national effort to combat this condition [7].

By being aware of the dental and oral characteristics linked to SCD and correlating them with systemic symptoms, pediatric and general dentists can contribute to early diagnosis. This one-of-a-kind study in India evaluates the awareness and knowledge of pediatric and general dentists regarding SCD, encompassing its systemic manifestations, diagnostic approaches, oral implications, and dental care strategies. The study revealed that pediatric dentists possess satisfactory knowledge about SCD, whereas general dentists demonstrated knowledge gaps.

Our study's findings are consistent with existing literature, which highlights significant knowledge gaps among healthcare professionals regarding SCD. For instance, Gomes et al. reported inadequate knowledge among physicians and nurses in Brazil, emphasizing the need for improved training in SCD management [10]. Similarly, Silva et al. found insufficient knowledge about blood disorders among undergraduate dentistry students [11].

In contrast, Meslet et al. reported satisfactory awareness levels among dental students [3]. However, our study's results align more closely with those of Segunmaru et al., who found that a large number of students lacked knowledge about SCD [12]. Druye's study also supports our findings, highlighting the importance of educational initiatives to enhance knowledge acquisition and promote high-quality care delivery among healthcare professionals [13].

The study by Luna et al. further reinforces our results, demonstrating that dental surgeons in Brazil struggled with knowledge gaps about SCD, with 57.1% citing lack of knowledge as a major difficulty [14].

Dental awareness of orofacial manifestations differs across regions due to variations in genetic inheritance patterns and the demographics of affected populations [15]. The persistent knowledge gaps underscore the need for targeted education and standardized guidelines to improve healthcare professionals' understanding and management of SCD. Notably, this study is a pioneering effort in India, providing unique insights into dental professionals' knowledge gaps. By highlighting these gaps, this research contributes to the growing awareness of the need for enhanced education and training in this field.

The dearth of research on this topic in India, combined with Madhya Pradesh's location in the country's sickle cell belt, underscores the significance and relevance of this study, particularly given the region's substantial disease burden. It offers valuable insights for refining dental care, guiding focused educational initiatives, and fostering collaborative healthcare strategies. By informing public health policies and practices, this study has the potential to positively impact patient care and outcomes.

Future directions of this research include expanding the study's scope, developing dental guidelines, implementing continuing education programs, fostering collaboration with hematologists and pediatricians, and investigating oral health outcomes to better serve affected populations.

Limitations

This research faced limitations, including its confinement to Madhya Pradesh, which restricted its geographical scope. The study's reliance on self-reported data and closed-ended questions may have limited insight depth. Additionally, the small sample size and non-probability sampling method (purposive and snowball sampling) could have introduced biases. Furthermore, the online survey distribution method through WhatsApp groups and personal networks may have excluded less digitally engaged dentists, potentially impacting the study's representativeness and generalizability.

## Conclusions

This pioneering research conducted in Madhya Pradesh, India, brings to light a substantial disparity in knowledge and understanding between pediatric dentists and general dentists concerning SCD and its profound implications for oral health. Notably, while pediatric dentists demonstrated a satisfactory level of knowledge about SCD management, general dentists exhibited significant knowledge gaps in critical areas directly impacting patient care, including SCD testing timing, bone marrow transplantation, oral manifestations, pulp changes, and local anesthesia use in SCD patients. These findings highlight the necessity for the implementation of targeted educational initiatives designed to enhance the knowledge and awareness of dental professionals, with a particular focus on general dentists. Equipping dental practitioners with accurate and comprehensive knowledge about SCD and its oral health implications can significantly enhance patient care, leading to early diagnosis in collaboration with hematologists and pediatricians, improved health outcomes, reduced complications, and an overall better quality of life for SCD patients. The study's findings highlight the need for collaborative efforts in dental education and public health policy to build a knowledgeable dental workforce, ultimately driving progress towards the national goal of eliminating SCD.

## Appendices

Appendix A 

**Table 3:** Questionnaire

Questionnaire		
(A) Questions based on demographic details		
1.	Email ID	
2.	Age (years)	
3.	Gender	
4.	Which of the following best describes you?	Pediatric dentist
		General dentist treating pediatric patients
		General dentist not treating pediatric patients
5.	Years of clinical experience?	Less than 5 years
		5 - 10 years
		More than 10 years
(B) Questions based on awareness about the association of SCD with oral health and the National Sickle Cell Anemia Elimination Mission		
6.	Do you think sickle cell disease is associated with oral health?	Yes
		No
		Maybe
7.	Are you aware of the National Sickle Cell Anemia Elimination Mission launched by P.M. from Shahdol, Madhya Pradesh, to eliminate SCD in India by 2047?	Yes
		No
(C) Questions based on basic knowledge about sickle cell disease		
8.	Is sickle cell disease an inherited blood disorder?	Yes
		No
		Maybe
9.	The lifespan of RBCs in sickle cell disease is reduced to?	10-20 days
		30-40 days
		60 days
10.	The appropriate time to test sickle cell disease in a child?	Newborn screening
		6 months to 10 years
		Adolescent screening
11.	Can sickle cell disease be diagnosed using a simple blood test?	Yes
		No
12.	Sickle cell disease can be cured by?	Radiotherapy
		Bone marrow transplant
		Cannot be completely cured
(D) Questions based on knowledge of SCD-related oral features and dental management strategies		
13.	Are you aware of oral manifestations of SCD?	Yes
		No
14.	If Yes, what are the common oral manifestations of SCD in children?	Delayed tooth eruption
		Changes in tongue and oral mucosa
		Enamel and dentin hypo-mineralization
		Dental caries
		All of the above
15.	Which of the following statements are correct about pulp changes in patient with SCD?	No pulp changes
		Pulp necrosis in otherwise clinically healthy teeth with/without pain
16.	Are Oral fungal infections more common in SCD?	Yes
		No
		Maybe
17.	Are radiographic bone changes evident in patients with SCD?	Yes
		No
		Maybe
18.	Is it recommended to give prophylactic antibiotic in patients with SCD before any surgical procedure like extraction of tooth?	Yes
		No
		Maybe
19.	Which analgesic is contraindicated in patients with SCD?	Morphine
		Fentanyl
		Aspirin
20.	Is local anesthesia contraindicated in patients with SCD?	Yes
		No
		Maybe
21.	Can anxiolytic drugs (benzodiazepine, hydroxyzine) and conscious sedation be used in patients with SCD to eliminate dental stress and anxiety?	Yes
		No
		Maybe
22.	Do you think preventive dental care is important in such patient?	Yes
		No
